# Beneficial effects of a high-anthocyanin diet versus a Westernized diet on colorectal cancer risk: a systematic review

**DOI:** 10.3389/fimmu.2026.1736018

**Published:** 2026-02-12

**Authors:** Alberto Vásquez, Paula Zúñiga, Keila Torres, Andrew F. G. Quest, Layla Simón

**Affiliations:** 1 Escuela de Nutrición y Dietética, Universidad Finis Terrae, Santiago, Chile; 2 Cellular Communication Laboratory, Center for Studies on Exercise, Metabolism and Cancer (CEMC), Interdisciplinary Nucleus of Biology and Genetics (NBG), Institute of Biomedical Sciences (ICBM), Faculty of Medicine, Universidad de Chile, Santiago, Chile; 3 Advanced Center for Chronic Diseases (ACCDiS), Faculty of Medicine, Universidad de Chile, Santiago, Chile

**Keywords:** antioxidants, bioactive compounds, colorectal cancer, dietary intervention, good health, western diet

## Abstract

**Introduction:**

Colorectal cancer (CRC) is one of the leading causes of morbidity and mortality worldwide. Its incidence has been strongly associated with dietary patterns of the Western diet (WD), which are characterized by high intakes of saturated fats, ultra-processed foods, red and processed meats, refined grains, and added sugars. In contrast, numerous studies have highlighted the potential health benefits of anthocyanins—bioactive compounds with anti-inflammatory, antioxidant, and antitumoral properties. This systematic review aimed to evaluate the effects of anthocyanin-rich diets on CRC prevention and compare them with the effects of WD.

**Methods:**

A systematic search was conducted in PubMed, Scopus, and SciELO databases following PRISMA 2020 guidelines. Search terms included: “anthocyanin” OR “anthocyanins” AND “prevention” AND “colorectal cancer” AND “Western diet” AND “effect.”

**Results:**

Sixteen studies met the inclusion criteria. Anthocyanin-rich interventions demonstrated consistent preventive effects, including the modulation of oxidative stress, suppression of oncogenic signaling pathways, induction of apoptosis in CRC cells, and restoration of gut barrier integrity and microbial diversity. Notably, these interventions exerted significant anti-inflammatory effects by downregulating pro-inflammatory cytokines, inhibiting NF-κB, and attenuating colitis-associated tumorigenesis. In contrast, Western dietary patterns were consistently associated with enhanced colonic inflammation, alterations in gut microbial composition, impairment of mucosal immune regulation, and increased colorectal tumor burden.

**Discussion:**

This systematic review provides evidence supporting the protective role of anthocyanin-rich diets in CRC prevention. However, further research is needed to examine drug-nutrient interactions in the context of comorbidities, evaluate various sources of anthocyanins, and better understand the factors influencing their bioavailability and absorption.

**Systematic Review Registration:**

https://osf.io/b56yz/overview, identifier osf-registrations-b56yz-v.

## 1 Introduction

Colorectal cancer (CRC) is a malignancy arising from adenomatous polyps in the colon or rectum that can evolve from benign lesions into invasive tumors. According to the Global Cancer Observatory (GLOBOCAN), CRC ranks as the third most frequently diagnosed cancer and the second leading cause of cancer-related mortality worldwide, accounting for over 900,000 deaths in 2022 (1). While CRC incidence increases with age, a notable upward trend in early onset CRC, defined as diagnosis before the age of 50, has been observed and indicates a shifting epidemiological trend (2, 3). Moreover, CRC is the leading cause of mortality and disability-adjusted life years (DALYs) attributable to dietary risks among individuals aged 15 to 49 worldwide (4).

CRC exhibits considerable molecular heterogeneity, with subtypes including microsatellite instability, chromosomal instability, and the CpG island methylator phenotype (5). Approximately 10 to 20% of CRC cases occur in individuals with a positive family history, and nearly 5% are attributable to hereditary cancer syndromes that can be identified through germline genetic testing. Most CRCs follow a well-characterized sequence of tumorigenesis involving the stepwise accumulation of mutations in *APC*, *KRAS*, and *TP53* (6). However, the majority of CRC cases are classified as sporadic. In such cases, risk is further elevated by the presence of longstanding inflammatory bowel disease and is significantly influenced by various factors, including the gut microbiome, age, genre, race, and socioeconomic status. Modifiable lifestyle and dietary factors also play a critical role, such as excessive alcohol consumption, cigarette smoking, obesity, physical inactivity, low dietary fiber intake, high consumption of red or processed meats, and diets low in calcium, vitamins, and dairy products (6–8).

Among these modifiable risk factors, adherence to a Western dietary (WD) pattern has emerged as a particularly concerning contributor. This dietary pattern is characterized by the frequent consumption of refined foods, such as white flour, white rice, and added sugars, along with high intake of animal protein, saturated fats, and ultra-processed foods, including processed meats, packaged snacks, and sugary beverages. Prolonged intake of WD disrupts physiological processes and impacts on health by promoting weight gain, dyslipidemia, altered energy metabolism, and immune system activation. These alterations have been consistently linked to the development of non-communicable diseases such as obesity, type 2 diabetes, and metabolic inflammation, which in turn increase the risk of cardiovascular disease, digestive disease, and cancer (9–12). Moreover, WD patterns have been shown to significantly exacerbate colitis, impair recovery from intestinal injury, and promote colorectal carcinogenesis by inducing mRNA signatures associated with inflammation, innate and adaptive immunity, B-cell and T-cell function, and antigen processing pathways (13).

In contrast, accumulating evidence highlights the potential protective effects of anthocyanins, natural polyphenolic compounds responsible for the red, purple, blue, and pink pigmentation found in many fruits and vegetables. Anthocyanins are a subclass of flavonoids synthesized via the phenylpropanoid pathway that primarily occur as glucosides of anthocyanidins. These compounds are stored in the vacuoles of plant cells. Common dietary sources include blackberries, raspberries, blueberries, grapes, plums, apples, red cabbage, and cauliflower (14, 15). Anthocyanins exhibit diverse bioactivities, including antioxidant, anti-inflammatory, anti-angiogenic, and anti-carcinogenic properties. Consequently, higher anthocyanin intake has been associated with a reduced risk of type 2 diabetes, cardiovascular disease, improved metabolic regulation in obesity, and neuroprotective effects (16–19).

Specifically in CRC, a significant inverse association has been reported between total anthocyanin intake and CRC risk (20). In addition, anthocyanins have been shown to exert pro-apoptotic effects and inhibit cell cycle progression, tumor cell invasion, and metastatic dissemination (21, 22). They also demonstrate significant protective effects against inflammation and increased intestinal permeability, while promoting colonic health through modulation of gut microbiota composition and microbial metabolic activity (23). Moreover, anthocyanins have shown potential to bind and inhibit immune checkpoint molecules such as PD-1 and PD-L1, thereby enhancing antitumor immune responses within the tumor microenvironment and promoting cancer cell death (24). Although several dietary polyphenols have been implicated in CRC prevention (25, 26), anthocyanins were selected as the focus of this review because their biological effects can be directly connected to key mechanisms underlying CRC pathogenesis. As previously mentioned, anthocyanins modulate inflammatory signaling, epithelial barrier integrity, immune responses, and gut microbiota composition within the colorectal environment (27), which supports their focused evaluation in the context of WD patterns and CRC risk.

Likewise, considering the rising global incidence of CRC and the widespread availability of anthocyanin-rich foods, this systematic review investigates the potential chemopreventive effects of a high-anthocyanin diet in comparison to a WD pattern. This analysis supports the hypothesis that increased consumption of anthocyanin-rich foods is inversely associated with CRC development, in contrast to the pro-inflammatory and tumor-promoting features typically attributed to WD patterns.

## 2 Methods

### 2.1 Information sources

This systematic review was conducted following the PRISMA 2020 guidelines to ensure the extraction of accurate and reliable information, as well as to promote transparency and comprehensiveness. The study was registered in the Open Science Framework (28). Two researchers independently performed the literature search during the first semester of 2024 and screened the retrieved articles based on titles, abstracts, and full texts. The search was conducted across multiple electronic databases, including PubMed, Scopus, and SciELO.

### 2.2 Search strategy

The PICO framework was used to guide the development of the research question. The Population (P) included adults at risk of developing colorectal cancer; the Intervention (I) was a high-anthocyanin diet; the Comparison (C) was a standard Western diet; and the Outcome (O) was the prevention of colorectal cancer. The formulated research question was: What evidence exists regarding the potential preventive effects of anthocyanin-rich dietary patterns compared with Western dietary patterns on colorectal cancer development when evaluating available *in vitro*, *in vivo*, and human studies? Keywords and search terms were defined in both English and Spanish to maximize the comprehensiveness of the search. These included: “anthocyanins,” “colorectal cancer,” “high-anthocyanin diet,” “prevention,” and “Western diet.”

Subsequently, search equations were constructed using Boolean operators, such as: “anthocyanin” OR “anthocyanins” AND “prevention” AND “colorectal cancer” AND “Western diet” AND “effect”.

### 2.3 Inclusion and exclusion criteria

The inclusion criteria for this review selected studies conducted in adults aged 18 years or older, animal or cell models of colorectal cancer; publications in English or Spanish; articles published from 2018 onward; and original research that specifically investigated anthocyanins and Western diets as the primary variables. Exclusion criteria discarded studies involving individuals with cancers other than colorectal cancer; articles published in languages other than English or Spanish; and non-original research, such as review articles. Only colorectal cancer (CRC) articles were included. Studies focusing exclusively on colon or rectal cancer were not included, even when the same cell lines or experimental models were used.

### 2.4 Data analysis

Data extracted from the selected studies included reported effects of anthocyanins, changes in gut microbiota composition, effects of anthocyanins on colorectal cancer cell proliferation, and outcomes associated with Western dietary patterns. Microsoft Excel was used to organize and manage the article selection process, including the identification of duplicate records and the screening of articles based on their titles and abstracts.

## 3 Results

**Figure 1:**
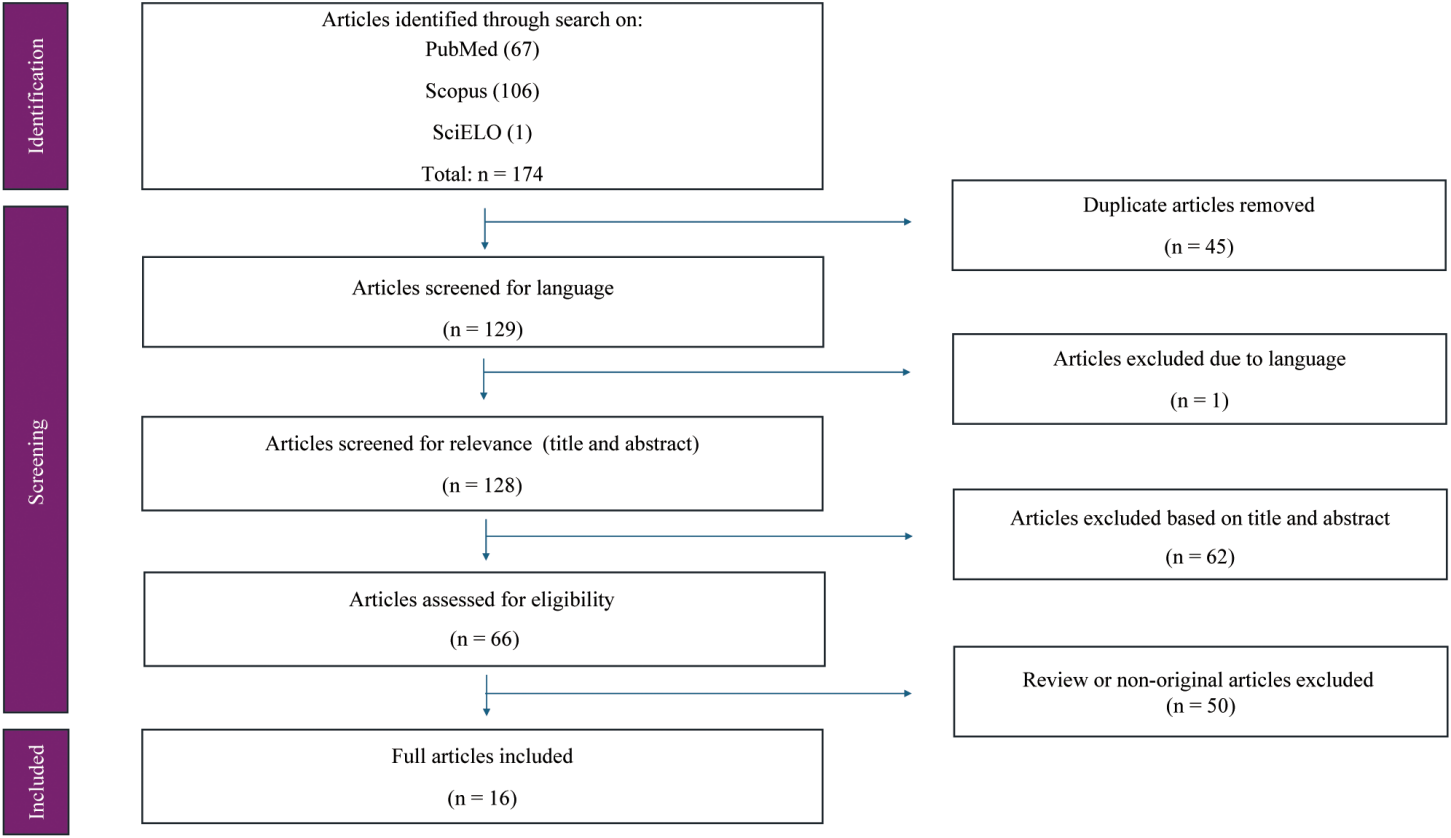
PRISMA flow diagram.

A total of 174 articles were identified during the initial search process. In the first screening phase, 45 duplicate records were removed. In the second phase, one article was excluded due to language, and 62 articles were excluded based on title and abstract evaluation. An additional 50 articles were excluded for not representing original research. Ultimately, 16 original articles met the inclusion criteria and were included in the final analysis (**Figure 1**).

The characteristics of the selected articles are summarized in **Table 1**. Among the experimental studies, three were conducted exclusively *in vitro* (29–31), three were conducted exclusively *in vivo* (13, 32, 33), three combined *in vivo* and *in vitro* approaches (34–36), and one study incorporated *in vivo*, *ex vivo*, and clinical trial methodologies (37). Additionally, six epidemiological studies were identified, including case-control (38–40), prospective cohort (41, 42), and nested case-cohort (43) designs. The studies were conducted across a diverse range of geographic regions, including Europe (Spain, Denmark, Italy, United Kingdom), Asia (China, Iran, Taiwan, Malaysia), and the Americas (United States of America, Colombia).

**Table 1:** Characteristics of studies included in the systematic review.

Year	First author	Title	Type of study	Country of study	Type of diets	Important results	Ref
2023	Abd Rashid	Dietary patterns associated with colorectal cancer risk in the Malaysian population: a case-control study with exploratory factor and regression analysis	Case- control	Malaysia	Allergenic diet; Plant-based diet; Processed diet, and energy dense diet	Processed diet high in confectionaries and fast foods was associated with increased CRC risk (OR = 3.45; 95% CI: 1.25-9.52)	(40)
2023	Tsai	Hibiscus Anthocyanins Extracts Induce Apoptosis by Activating AMP-Activated Protein Kinase in Human Colorectal Cancer Cells	*In vitro*	Taiwan	*Hibiscus sabdariffa L.* (HAs), including anthocyanins	HAs extracts reduced 10-80% cell viability and induced apoptosis in CRC by activating AMPK, inhibiting Akt, and increasing Fas/Fas L to produce intrinsic and extrinsic apoptosis	(31)
2023	Chen	Sirtuin1 (SIRT1) is involved in the anticancer effect of black raspberry anthocyanins in colorectal cancer	*In vivo* and *in vitro*	China	Black raspberry (BRB) anthocyanins	Dietary supplementation with BRB anthocyanins suppresses 50% CCR progression by downregulating NLRP3-mediated inflammation, potentially via SIRT1 activation, NOX2 inhibition, and reduced ROS production	(36)
2022	Castelló	Adherence to the Western, Prudent and Mediterranean Dietary Patterns and Colorectal Cancer Risk: Findings from the Spanish Cohort of the European Prospective Investigation into Cancer and Nutrition (EPIC-Spain)	Prospective cohort	Spain	Western, Prudent, Mediterranean	High adherence to a Mediterranean dietary pattern and low adherence to a Western diet were significantly associated with a reduced risk of CRC (HR_1SD-increase_ = 1.17; 95% CI: 0.99-1.37)	(42)
2022	May	Modification of Diet to Reduce the Stemness and Tumorigenicity of Murine and Human Intestinal Cells	*In vivo*, *ex* *vivo*, and Clinical Trial	United Kingdom	Black raspberry (BRBs)- supplemented diet	Black raspberry diet reduced intestinal stem cell numbers, delayed tumor progression in Apc-mutant models, decreased tumorigenicity in mouse and human CRC cells, and doubled survival time	(37)
2022	Speciani	Flavonoid Intake in Relation to Colorectal Cancer Risk and Blood Bacterial DNA	Case- control	Italy	Anthocyanidins and flavanones	Intake of anthocyanidins (OR = 0.24; 95% CI: 0.11-0.52) and flavanones (OR = 0.18; 95% CI: 0.08-0.42) was significantly associated with a decreased risk of colorectal cancer and was correlated with alterations in circulating bacterial DNA, implying a potential modulation of intestinal permeability	(39)
2022	Rodríguez	Dietary Supplementation with Black Raspberries Altered the Gut Microbiome Composition in a Mouse Model of Colitis-Associated Colorectal Cancer, although with Differing Effects for a Healthy versus a Western Basal Diet	*In vivo*	United States of America	Western diet/Black raspberry (BRB) (5–10% w/w)	BRB supplementation significantly altered the gut microbiome and reduced colon tumor development to levels similar to the control group	(33)
2021	Feng	Intake of processed meat, but not sodium, is associated with risk of colorectal cancer: Evidence from a large prospective cohort and two-sample Mendelian randomization	Prospective cohort	United Kingdom	Western Diet	Processed - meat intake is associated with an increased risk of CRC (HR 1.23; 95% CI: 1.03-1.46)	(41)
2021	Mudd	Berry anthocyanins inhibit intestinal polyps and colon tumors by modulation of Src, EGFR and the colon inflammatory environment	*In vivo* and *in vitro*	United States of America	Anthocyanin mixture from bilberry (Anthos)	Bilberry-derived anthocyanidins significantly inhibit CRC cell proliferation and reduce 50% tumor burden, associated with suppression of Src and EGFR phosphorylation and modulation of the inflammatory microenvironment	(34)
2021	Yu	*Aronia melanocarpa* Elliot anthocyanins inhibit colon cancer by regulating glutamine metabolism	*In vivo* and *in vitro*	China	*Aronia melanocarpa* Elliot anthocyanins (AMA)	AMA inhibit colon cancer development in a dose-dependent manner by reducing inflammatory cytokine secretion and suppressing the mTORC1 signaling pathway through downregulation of GLS and SLC1A5	(35)
2020	Benninghof	Consumption of the Total Western Diet Promotes Colitis and Inflammation-Associated Colorectal Cancer in Mice	*In vivo*	United States of America	Western Diet	Western-style diet exacerbated colitis, delayed recovery, and increased colon tumorigenesis twice compared to the control group; effect mitigated by calcium and vitamin D supplementation	(13)
2020	Wei	Anthocyanins from *Aronia melanocarpa* Induce Apoptosis in Caco-2 Cells through inhibition of the Wnt/β-Catenin Signaling Pathway	*In vitro*	China	Anthocyanin extract (*Aronia melanocarpa*)	Anthocyanins induced cell cycle arrest and apoptosis in CRC cells (in a dose-dependent manner) via inhibition of the Wnt/β-catenin pathway, reducing β-catenin and related proteins	(30)
2019	Andersen	Intake of Red and Processed Meat, Use of Non-Steroid Anti-Inflammatory Drugs, Genetic Variants and Risk of Colorectal Cancer: A Prospective Study of the Danish “Diet, Cancer and Health” Cohort	Nested case- cohort	Denmark	Red and processed meat/non-steroid anti-inflammatory drug (NSAID) use	Significant associations were observed between red and processed meat intake and polymorphisms in genes related to fatty acid metabolism (HR = 1.05; 95% CI: 0.98–1.13)	(43)
2019	Bahrami	Dietary intake of polyphenols and risk of colorectal cancer and adenoma: A case-control study from Iran	Case- control	Iran	Polyphenol- rich (anthocyanin, flavonoids, phenolic acids)	Higher intake of total polyphenols (OR = 0.05; 95% CI: 0.01−0.19), anthocyanins (OR = 0.21; 95% CI: 0.08−0.55), and flavonoids (OR = 0.36; 95% CI: 0.16−0.79) was associated with decreased risk of CRC and adenoma	(38)
2019	Zapata	Vinegar of Andean berries (*Vaccinium meridionale* SW): Antioxidant and antiproliferative activity in colon cancer cells SW480	*In vitro*	Colombia	Andean Berries (*Vaccinium meridionale)* (Polyphenols, anthocyanins and hydroxycinnamic acids)	The vinegar showed high antioxidant activity and significantly inhibited the proliferation of CRC cells in a dose-dependent manner, indicating potential chemopreventive properties	(29)
2018	Fernández	Functional Anthocyanin-Rich Sausages Diminish Colorectal Cancer in an Animal Model and Reduce Proinflammatory Bacteria in the Intestinal Microbiota	*In vivo*	Spain	Anthocyanins (mixture of dehydrated blackberries and strawberries)	Anthocyanin- enriched sausages reduced colon tumors by half, increased plasma antioxidant activity, and lowered pro-inflammatory bacteria *Bilophila wadsworthia*	(32)

Anthocyanins, or anthocyanin-rich sources, were the most frequently examined dietary components, reported in 11 of the 16 studies included in this review (29–39). These sources included blackberries, bilberries, black raspberries, *Aronia melanocarpa*, and *Vaccinium meridionale*, all of which demonstrated antioxidant, anti-inflammatory, and antiproliferative effects in CRC models. In contrast, the Western dietary pattern was consistently associated with an increased risk of colorectal cancer (13, 33, 40–43).

## 4 Discussion

CRC continues to rise in incidence globally, with growing evidence linking this trend primarily to dietary habits and lifestyle factors (13, 40–42, 44). In this context, the present systematic review compared the potential protective effects of high-anthocyanin dietary patterns with the harmful effects associated with Westernized diets in CRC risk.

### 4.1 Pathways modulated by anthocyanins in the prevention and progression of CRC

**Figure 2:**
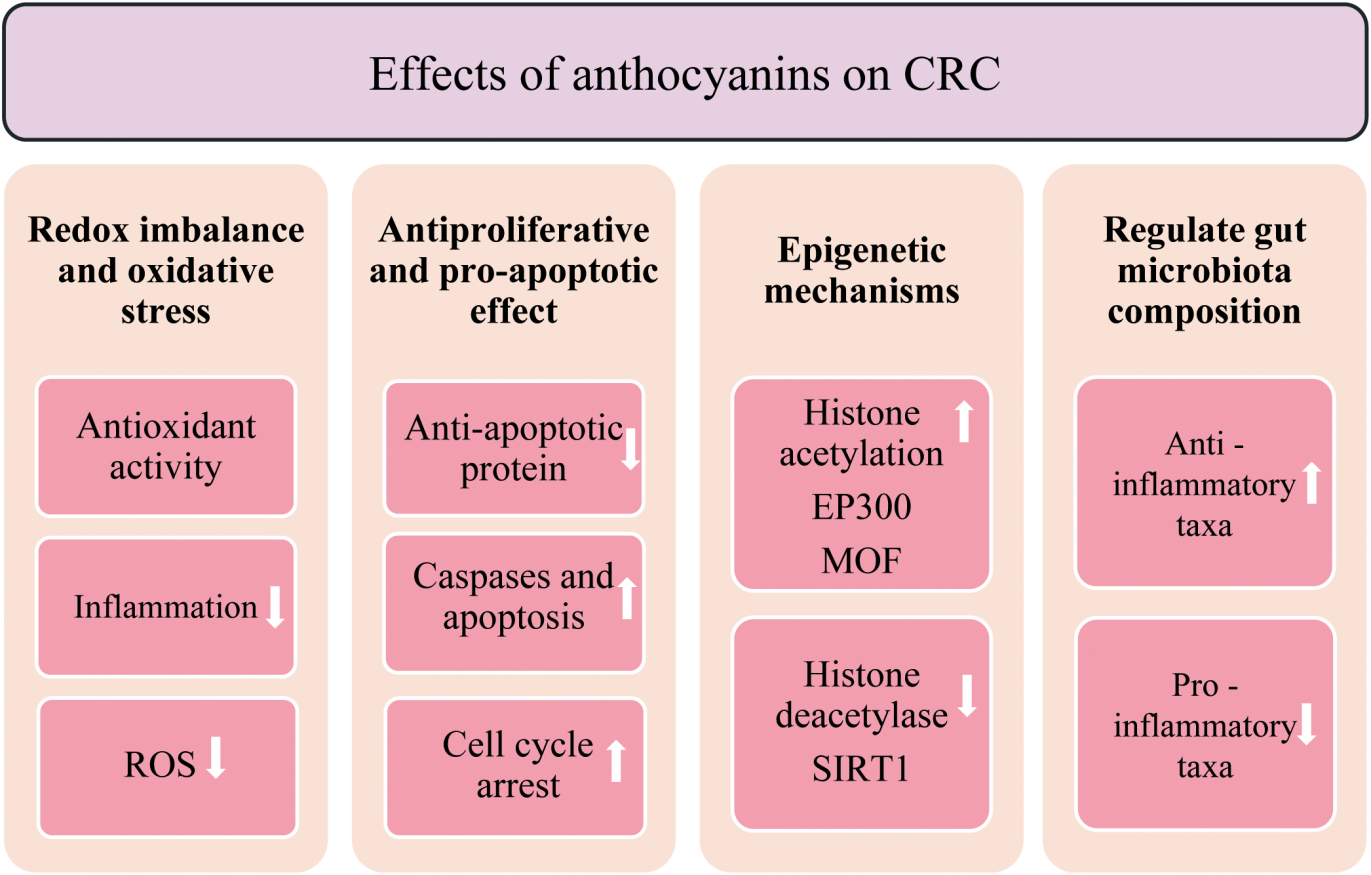
Role of anthocyanins in the inhibition of CRC progression. ↑ Increase; ↓ Decrease.

An increasing body of evidence suggests that anthocyanins suppress the initiation and progression of CRC by modulating oxidative stress, inflammatory responses, epigenetic regulation, and gut microbiota composition (**Figure 2**). Chen et al. (2023) demonstrated that black raspberry (BRB) anthocyanins reduced colorectal tumor development in AOM-induced mouse models and CRC cell lines by modulating histone acetylation. Specifically, BRB anthocyanin treatment led to a global increase in histone acetylation, significantly upregulating the expression of histone acetyltransferases EP300 and MOF, while downregulating the expression of the histone deacetylase SIRT1. This resulted in elevated acetylation levels at histone H4 lysine residues H4K5, H4K12, and H4K16. In addition, BRB anthocyanins downregulated the expression of the anti-apoptotic protein Bcl-2 and cell cycle regulators c-Myc and cyclin D1, while upregulating the pro-apoptotic protein Bax. These molecular changes were associated with activation of the NF-κB signalling pathway, suggesting that BRB anthocyanins promote apoptosis and cell cycle arrest in CRC cells (36). On the other hand, the Andean berry *Vaccinium meridionale* and its derivatives are recognized for their high content of antioxidant bioactive compounds. However, the fermentation process influences the composition of polyphenols, particularly reducing anthocyanin levels. In comparative analyses, vinegar produced from this berry shows lower antioxidant activity than the original juice, while the alcoholic beverage demonstrates a significantly greater antioxidant effect. Additionally, the vinegar has been shown to inhibit cell proliferation in a concentration-dependent manner in SW480 colon cancer cells (29).

An anthocyanidin mixture derived from bilberry (Anthos) has been reported to suppress the formation of intestinal polyps and tumors in a CRC mouse model. *In vivo*, anthocyanidin treatment significantly reduced the phosphorylation of Src, EGFR, STAT5, and STAT3, and was accompanied by a reduction in inflammation (34). Similarly, Yu et al. (2021) reported that anthocyanins from *Aronia melanocarpa* (AMA) exhibit anticancer effects in both *in vitro* and *in vivo* CRC models. AMA inhibited Caco-2 cell proliferation and significantly reduced the expression of glutaminase (GLS) and SLC1A5, particularly at 40 mg/kg. This was accompanied by downregulation of mTORC1 signaling proteins, including p-p70S6K, p70S6K, ULK1, p-mTOR, 4EBP1, and p-4EBP1. In a CRC mouse model, AMA similarly suppressed these markers, decreased α-ketoglutarate levels, and reduced tumor malignancy. These findings suggest that AMA inhibits tumor progression by disrupting glutamine metabolism and mTORC1 pathway activation (35). On the other hand, Wei et al. (2020) reported that treatment with AMA reduced cell viability and increased cell death in Caco-2 cells by inducing apoptosis and blocking S-phase progression in a concentration-dependent manner. AMA downregulated the expression of Survivin, c-Myc, and Cyclin D1, while upregulating pro-apoptotic markers such as Bax, cleaved caspase-3, and cytochrome c. Bcl-2 expression was significantly decreased. AMA also promoted the degradation of intracellular free β-catenin without affecting its transcription or translation. This effect was mediated by inhibition of GSK3β phosphorylation, thereby precluding signaling via the Wnt/b-catenin pathway (30).

Tsai et al. (2023) reported that anthocyanin extracts from *Hibiscus sabdariffa* induce apoptosis in CRC cells by upregulating pro-apoptotic Bcl-2 family members (tBid, Bax, Bad), increasing the levels of apoptosis-inducing factor (AIF) and cytochrome c levels, and reducing Bcl-xL expression. They also activated the Fas/FasL pathway and downstream caspases (3, 8, 9, and PARP), while disrupting mitochondrial membrane potential and inhibiting Akt signaling (31). Likewise, May et al. (2022) demonstrated that dietary supplementation with BRBs increased survival in Apc-deficient mice by reducing intestinal stem cell (ISC) marker expression and tumorigenic potential. Similar effects were observed in human CRC organoids, suggesting that anthocyanins modulate ISC homeostasis to exert chemopreventive effects (37). Moreover, epidemiological evidence has shown a strong inverse association between dietary anthocyanin intake and CRC risk (OR = 0.21; 95% CI: 0.08–0.55) (38).

Anthocyanins from AMA inhibit superoxide dismutase (SOD) activity in Caco-2 cells. When treated with 50, 100, and 200 micrograms per milliliter of anthocyanins, SOD levels progressively decreased from 46.2 units per milligram to 20.0 units per milligram, reducing the cellular capacity to neutralize oxygen-free radicals and intensifying lipid peroxidation and oxidative stress. Moreover, malonic dialdehyde levels, which reflect the extent of lipid peroxidation and indirectly indicate cellular damage, increased significantly. These findings suggest that anthocyanins may inhibit SOD activity, impair the scavenging of oxygen-free radicals, exacerbate oxidative stress and lipid peroxidation, and ultimately induce apoptosis in CRC cells (30).

### 4.2 Changes in gut microbiota composition

Four studies assessed the effects of anthocyanins on gut microbiota. Rodriguez et al. (2022) examined the impact of freeze-dried BRB supplementation in a mouse model of colitis-associated colorectal cancer. Supplementation with 5% or 10% (w/w) whole BRB favorably modulated the fecal microbiome, particularly during active colitis, and reduced both colitis severity and tumor burden in mice fed a total WD. BRB intake increased alpha diversity (i.e., species richness, evenness, or diversity within the sample) and significantly altered beta diversity (i.e., the similarity between two or more communities), reflecting broad changes in microbial community structure. Distinct microbial profiles were observed before colitis onset, during inflammation, and throughout tumor development. The intervention led to increased relative abundance of bacterial families such as Lachnospiraceae, Ruminococcaceae, and Rikenellaceae, with variations depending on the basal diet and disease phase. Of particular interest, BRB supplementation enriched *Bifidobacterium pseudolongum*, a taxon linked to anti-inflammatory and immunomodulatory effects (33).

A related study by Fernández et al. (2018) evaluated the effects of anthocyanin-enriched functional sausages on gut microbiota composition in rats over a 20-week period. Animals were assigned to one of three dietary groups: standard, control sausages, or functional sausages supplemented with 0.1% (w/w) anthocyanins derived from a mixture of dehydrated blackberries and strawberries. Metagenomic analysis revealed that, at the genus level, the only statistically significant difference was found within the Desulfovibrionaceae family, specifically in the relative abundance of *Bilophila wadsworthia*, a sulfite-reducing bacterium known for producing hydrogen sulfide (H_2_S). This species accounted for 7.90% of the total metagenomic content in the control sausage cohort, but was reduced to 4.94% in the functional sausage group. These findings suggest that dietary anthocyanins may influence gut microbiota composition by reducing the relative abundance of potentially pro-inflammatory taxa such as *Bilophila wadsworthia*, which may contribute to improved intestinal health (32). In a case-control study, Speciani et al., 2022 analyzed circulating bacterial DNA profiles in blood samples from CRC patients and matched controls, alongside dietary flavonoid intake. They found that anthocyanins were negatively associated with operational taxonomic units (OTUs) assigned to Flavobacterium and Legionella, and positively associated with OTUs assigned to the Brevundimonas genus. Additionally, anthocyanidins showed a negative association with the Escherichia–Shigella group and a positive association with OTUs from the Oligoflexales order. The observed associations imply that anthocyanins can modulate circulating bacterial DNA levels, possibly through effects on intestinal permeability (39). On the other hand, anthocyanins-treated *ApcMin*^/+^ mice inoculated with enterotoxigenic *Bacteroides fragilis*, a model of bacteria-induced inflammatory bowel disease that secretes a metalloprotease enterotoxin, showed a clear dose-dependent reduction in colon tumor counts compared to untreated controls (34).

### 4.3 Modulation of inflammatory pathways by anthocyanins

**Figure 3:**
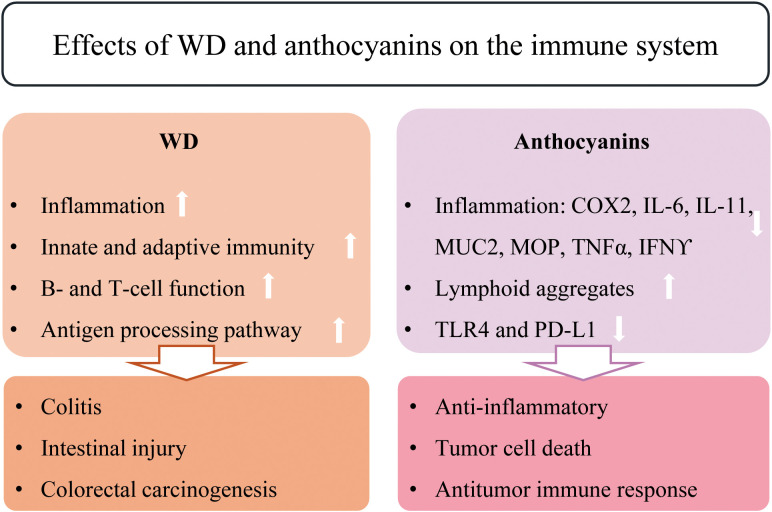
Effects of WD and anthocyanins on the immune system.

Several studies have demonstrated that anthocyanin-rich extracts modulate key inflammatory pathways and cytokine expression both *in vitro* and *in vivo*. BRB anthocyanins suppress inflammation in CRC models by downregulating SIRT1, which enhances NF-κB/p65 acetylation and reduces the expression of pro-inflammatory targets such as NOX2 and NLRP3. This effect is accompanied by a reduction in reactive oxygen species (ROS) levels (36). Additionally, anthocyanins have been shown to significantly reduce inflammatory markers, including COX-2, IL-6, IL-17, MUC2, MPO, TNF-α, and IFN-γ in CRC cells (**Figure 3**) (35). Berry anthocyanins have also been shown to modulate immune responses within the tumor microenvironment by inducing lymphoid aggregates and regulating key molecular markers. In adjacent normal tissue, berry anthocyanins reduced the expression of COX-2, meanwhile increasing the expression of IFN-γ, phospho-p38, TLR-4, and PD-L1. In the tumor tissue microenvironment, berry anthocyanins reduced levels of COX-2, TLR-4, and PD-L1, indicative of their chemopreventive modulation of the inflammatory environment in CRC (34).

### 4.4 Studies evaluating anthocyanin dosages

Across the revised studies, varying concentrations of anthocyanins were administered to CRC cell lines, including Caco-2, SW480, LoVo, HCT 116, and HT-29, as well as to organoids ISO48 and ISO50, with doses ranging from 20 to 3000 μg/mL (**Table 2**). All studies reported a consistent dose-dependent response, whereby higher anthocyanin concentrations correlated with greater anticancer effects.

**Table 2:** Anthocyanin concentrations used in *in vitro* studies.

CRC cell lines or human CRC organoids	Dosage	Reference
Caco-2	25, 50, 100, 200, and 300 μg/mL	(30)
HCT 116 and HT-29	Up to 200 μg/mL	(34)
LoVo	1000, 2000, and 3000 μg/mL	(31)
SW480 and Caco-2	25 and 50 μg/mL	(36)
SW480	20, 40, 80, 100, and 200 μg/mL	(29)
Caco-2	50, 100, 150, 200, and 250 μg/mL	(35)
ISO48, ISO50 and Caco-2	0.48 to 500 μg/mL	(37)

The anthocyanin dosages used in both *in vivo* and human studies are summarized in **Table 3**. In animal models, anthocyanins were administered either at defined doses based on body weight (mg/kg) or as anthocyanin-enriched diets. Human data, derived from both clinical trials and observational studies, involved dietary supplementation or intake estimation using validated food frequency questionnaires. In all cases, anthocyanin consumption was quantified to evaluate its potential role in the prevention and progression of CRC. Notably, these studies consistently reported lower anthocyanin intake among CRC patients compared to healthy controls, suggesting a potential protective effect.

**Table 3:** Anthocyanin dosages used in *in vivo* studies and in clinical and observational human research.

Sample size	Dosage	Reference
In vivo		
C57BL/6J *ApcMin*^/+^; 4 to 8 per group	- 20 mg/kg Anthos, administered five times per week for 4 weeks. - 40 mg/kg Anthos, administered three times per week for 4 weeks. - Anthos administered at three doses: low (20 mg/kg, three times per week ≈ 8.6 mg/kg/day), medium (40 mg/kg ≈ 17.1 mg/kg/day), and high (80 mg/kg ≈ 34.3 mg/kg/day).	(34)
C57BL/6J; 10 per group	7.0 μmol/g (4.1 g/kg) BRB anthocyanins. (≈ 16.4–24.6 mg per mouse per day for 45 days).	(36)
C57BL/6J mice with an inducible *Apcfl* mutation in either the ISC (*Lgr5CreERT2*) or intestinal crypt (*AhCre/VillinCreERT2*); 3 to 6 per group	10% BRB-supplemented diet (≈85 g freeze-dried BRB; ≈0.9 kg fresh BRB) two weeks prior to Apc loss and continuing until the end of the experimental study.	(37)
Fischer 344 rats; 10 per group	20 g/day of functional sausages containing 1.1 g anthocyanins/kg throughout the experimental period.	(32)
C57BL/6J mice; 32 per group	Dietary supplementation with 5 to 10% BRB(≈7–13 mg anthocyanins/day/mouse) for the duration of the experimental study.	(33)
C57BL/6 mice; 13 per group	AMA (20 or 40 mg/kg) daily starting on day 7 of the recovery period and continuing for approximately 4 months.	(35)
Clinical and observational evidence in humans		
10 CRC patients	20 g freeze-dried berry powder was mixed with 100 mL of water and consumed orally three times a day (60 g/day for 1–9 weeks).	(37)
100 CRC cases and 200 controls, of which 100 had intestinal adenoma (IA), and 100 were healthy controls	An *ad hoc* food composition database was developed using flavonoid content from a food frequency questionnaire to quantify subclass intake, including anthocyanins. Mean anthocyanin intake was 28.3 mg/day (SD 32.3). A higher proportion of controls were in the highest intake tertile (>30.62 mg/day), while most CRC cases were in the lowest tertile (≤9.45 mg/day).	(39)
129 CRC, 130 colorectal adenoma cases, and 240 healthy controls	Polyphenol intake, including anthocyanins, was estimated using a validated semi-quantitative food frequency questionnaire. Anthocyanin intake (mg/day): controls – 23.7 (16.2–34.4), CRC – 15.7 (8.7–25.8), adenoma – 18.8 (10.3–34.0).	(38)

Anthos, Anthocyanidin mixture; BRB, Black raspberries; AMA, Aronia melanocarpa Elliot anthocyanins.

### 4.5 Effects of western dietary patterns on CRC

Among the 16 studies included in this systematic review, six provided clear evidence linking the WD pattern to the development of CRC (13, 33, 40–43).

Andersen et al. (2019) identified significant gene–diet interactions between red and processed meat intake and four polymorphisms (CCAT2 rs6983267, TP53 rs1042522, LPCAT1 rs7737692, and SLC25A20 rs7623023) associated with CRC risk in a Danish cohort. T-allele carriers of CCAT2 rs6983267 had a lower CRC risk compared to GG homozygotes, while variant alleles of TP53 rs1042522 and LPCAT1 rs7737692, as well as the AA genotype of SLC25A20 rs7623023, were linked to increased CRC risk with higher meat intake. These findings suggest that red and processed meats may promote CRC through pathways related to inflammation and fatty acid metabolism (43). Similarly, Feng et al. (2021) confirmed a causal relationship between processed meat consumption and a 23% increased CRC risk, independent of confounders such as sodium intake (41).

Preclinical studies demonstrated that mice fed with WD exhibited aggravated colitis, heightened inflammation and mucosal damage, delayed epithelial repair, and a significantly increased tumor burden compared to controls. These effects were attributed to nutritional deficiencies in the WD, particularly reduced levels of calcium and vitamin D (13). Further supporting this, Rodríguez et al. (2022) showed that WD-fed mice displayed altered gut microbiome composition and elevated tumor multiplicity, with WD exposures modulating key microbial taxa (33). In epidemiological studies, Castelló et al. (2022) observed a positive association between adherence to a WD pattern and CRC risk, particularly during the first 10 years of follow-up. Individuals in the highest quartile of WD adherence had a 53% increased risk of CRC, with the effect being especially pronounced for rectal cancer (42).

Abd Rashid et al. (2023) identified a processed dietary pattern rich in fast foods and confectionery that significantly increased the risk of CRC in a Malaysian population with diverse socioeconomic backgrounds (40). Together, these findings reveal that WD components not only promote local intestinal inflammation and dysbiosis but may also interact with host genetics and micronutrient deficiencies to drive colorectal tumorigenesis.

### 4.6 Inflammatory effects of WD

Emerging evidence highlights the critical role of WD components in the development of CRC, primarily through mechanisms involving chronic intestinal inflammation and gut microbiota dysregulation (13, 33). In a controlled animal study, transcriptomic analysis showed marked upregulation of genes linked to interferon response, inflammation, immunity, and chemokine signaling in mice fed a total WD during active colitis. These alterations, along with activation of B-cell and antigen processing pathways, persisted throughout recovery and paralleled ongoing mucosal injury. These findings suggest that sustained inflammation and colonic dysplasia are key mechanisms by which the total WD promotes colitis-associated colorectal carcinogenesis (13). Furthermore, genetic factors may modulate CRC risk under inflammatory conditions; for example, the TP53 rs1042522 polymorphism was shown to interact with nonsteroidal anti-inflammatory drug use, with GG homozygotes experiencing reduced CRC risk and carriers of the variant C allele showing increased risk (43). Collectively, these studies support the conclusion that WD patterns exacerbate colonic inflammation, which plays a central role in initiating and promoting tumorigenesis in the colon.

In a complementary study, Rodríguez et al. (2022) reported that although BRB supplementation altered gut microbiota composition and enhanced microbial diversity, it did not lead to consistent reductions in colonic inflammation in mice fed with WD. Histopathological analyses revealed persistent mucosal injury and inflammatory cell infiltration across groups, with significantly higher inflammation and mucosal damage scores in total WD-fed mice compared to those on the healthy diet. These findings suggest that the pro-inflammatory effects of the WD may override the potential anti-inflammatory benefits of dietary interventions such as BRB supplementation (33). Collectively, the evidence points towards the existence of distinct mechanistic pathways through which WD patterns and anthocyanin-enriched interventions influence colorectal carcinogenesis (**Table 4**). Notably, their divergent effects on intestinal inflammation, microbiota composition and oncogenic signaling provide critical insights into the dietary modulation of CRC risk and offer a framework for evaluating the preventive potential of bioactive nutritional compounds.

**Table 4:** Impact of anthocyanin-rich diet versus WD on CRC development.

Aspect	Anthocyanin-rich diet	Western diet
Composition	Includes blackberries, strawberries, Andean Berries (*Vaccinium meridionale)*, *Aronia melanocarpa*, *Hibiscus sabdariffa L.*, bilberry, black raspberries, and blueberries.	Characterized by high intake of red and processed meats, saturated fats, ultra-processed foods, refined grains, sugary snacks, full-fat dairy; low fiber intake; often accompanied by alcohol and tobacco use.
Effects on CRC cells	Inhibits cell proliferation and induces apoptosis.	Promotes cell proliferation and colonic adenoma formation.
Effects on inflammation and oxidative stress	Demonstrates antioxidant and anti-inflammatory activity by neutralizing free radicals and reducing oxidative stress.	Induces chronic inflammation and increases reactive oxygen species, leading to DNA damage.
Effects on gut microbiota	Reduces pro-inflammatory gut microbial populations.	Alters microbiota composition toward a pro-inflammatory profile
Overall effect on CRC	Prevents tumor initiation and progression.	Facilitates tumor development and growth.

## 5 Conclusions

### 5.1 Current state of knowledge

Although our review focused on studies published before mid-2024, recent research has provided additional insights that further strengthen and expand our understanding of how WD patterns and anthocyanin-rich diets influence CRC. Among CRC survivors in the United States, adherence to a WD pattern has been associated with poorer social functioning (45). Moreover, recent evidence confirms that WD, characterized by high intakes of trans fats and saturated fatty acids, is linked to an increased risk of CRC (46). Red meat consumption may promote CRC progression through the incorporation of N-glycolylneuraminic acid into intestinal epithelial cells, which activates the Wnt/β-catenin signaling pathway independently of immune-mediated mechanisms (47). In addition, the CRC Microbial Dietary Score (CMDS) has been positively associated with consumption of highly processed foods, such as processed meats, energy drinks and snacks, and negatively associated with intake of fiber-rich foods, including fruits, nuts, dark yellow vegetables, and legumes. Elevated CMDS correlated with microbial species enriched in CRC and was linked to a higher risk of CRC, particularly in tumors containing *F. nucleatum*, pks + *E. coli*, and enterotoxigenic *Bacteroides fragilis* (48).

On the other hand, anthocyanin and polyphenol-rich chokeberry and blueberry pomace extracts inhibit proliferation and metastasis in CRC cells by modulating ERK, Akt1, gp130, and STAT3 pathways (49). Similar results were observed for cyanidin 3 O glucoside (Cy3g), a natural anthocyanin, that dose dependently inhibited proliferation, migration, and invasion, while promoting apoptosis in CRC cells. In tumor-bearing mice, Cy3g reduced CRC growth by activating KLF4 and modulating the ERK and p38 signaling pathways (50). In addition, supplementation with 5% or 10% dehydrated calyces of Hibiscus sabdariffa, a significant source of anthocyanins and phenolic compounds, modified gut microbiota composition by increasing butyrate producing bacteria. These dietary interventions also elevated caspase 3 and cMyc expression, indicating potential activation of apoptotic mechanisms (51).

Despite encouraging evidence, the translation of anthocyanins into routine clinical practice remains limited by low oral bioavailability, extensive metabolism, and rapid systemic clearance, resulting in highly variable systemic exposure (52). In addition, substantial variability in anthocyanin content across food sources, processing methods, and formulations complicates standardization and reproducibility in clinical studies. Although bioavailability may be enhanced through advanced delivery systems, such as nanoencapsulation, nanogels, nanoemulsions, liposomal carriers, and chemical modifications, including acylation and co-pigmentation (53), evidence indicates that anthocyanins consumed within whole foods exhibit superior bioavailability and biological activity compared with isolated or purified extracts, likely due to synergistic interactions within the food matrix (54).

### 5.2 Limitations and future considerations

This systematic review provides valuable insights to the influence of anthocyanin intake and WD patterns on CRC risk; however, several limitations warrant consideration. Substantial heterogeneity across study designs, intervention durations, and the specific sources of anthocyanins and WD components introduces variability that limits direct comparison and synthesis. Moreover, the predominance of preclinical and observational studies restricts the ability to draw definitive causal inferences or to generalize findings to clinical settings. Additionally, the predefined terminology used in the search strategy, although considered the most relevant, did not include all possible synonyms or variations employed in CRC research, which may have reduced the retrieval sensitivity of the systematic search. Future systematic reviews should perhaps consider incorporating a broader and more comprehensive terminology to ensure the identification of additional relevant studies.

### 5.3 Conclusions

This systematic review highlights the contrasting effects of WD patterns and anthocyanin-rich diets in the development and prevention of CRC. WD, characterized by high intakes of red and processed meats, refined carbohydrates, and saturated fats, is consistently associated with chronic colonic inflammation, disruption of the gut microbial environment, and accelerated tumor progression. In contrast, anthocyanins exert multiple chemopreventive effects, including the reduction of oxidative stress, suppression of proinflammatory cytokine production, inhibition of oncogenic signaling pathways, such as those involving Wnt/β-catenin, mTORC1, and NF-κB, and restoration of intestinal barrier function and microbial diversity. Evidence from *in vitro*, *in vivo*, and human observational studies supports the notion of a dose-dependent inverse relationship between anthocyanin intake and CRC risk. Although anthocyanins are not intended to replace standard therapies, they may serve as complementary agents that enhance treatment response and modulate cancer-promoting pathways. These findings support anthocyanin-rich dietary strategies as a practical, biologically relevant, and accessible approach to CRC prevention. Further clinical trials are essential to validate their efficacy, clarify underlying mechanisms, and improve their bioavailability in human populations.

## Data Availability

The original contributions presented in the study are included in the article/**Supplementary Material**. Further inquiries can be directed to the corresponding authors.
